# Effects of the Design of Overview Maps on Three-Dimensional Virtual Environment Interfaces

**DOI:** 10.3390/s20164605

**Published:** 2020-08-16

**Authors:** Chien-Hsiung Chen, Meng-Xi Chen

**Affiliations:** Department of Design, National Taiwan University of Science and Technology, Taipei 10607, Taiwan; cchen@mail.ntust.edu.tw

**Keywords:** overview map, spatial perception, virtual environment, interface design

## Abstract

This study examined how users acquire spatial knowledge in an onscreen three-dimensional virtual environment when using overview maps. This experiment adopted a three (the size of overview maps) x two (the transparency of overview maps) between-subjects design. Three levels of the size of overview maps were evaluated, i.e., 1/2, 1/8, and 1/16 screen size. Comparisons between 20% transparent and 80% transparent were made. We asked 108 participants to complete spatial perception tasks and fill out questionnaires regarding their feelings. The results indicate the following: (1) The effects of the transparency of overview maps on users’ spatial perception vary with the size of overview maps. The 80% transparent overview map is significantly more efficient than the 20% transparent overview map in the condition of 1/2 screen size. However, the result is opposite in the condition of 1/8 screen size. (2) Users like the 80% transparent overview map significantly better than the 20% transparent overview map in the condition of 1/2 screen size. (3) Concerning subjective evaluations of satisfaction, preference, and system usability, overview maps in the condition of 1/8 screen size are significantly better than those in the condition of 1/2 screen size.

## 1. Introduction

As three-dimensional virtual environment (3D VE) has become more widely used in recent years, the usability, interactivity, and immersion of VE interfaces are expected to be better. Compared to the real environment, spatial perception in VEs has been found much less accurate, especially in perceptual judgments of distance and size [1,2,3]. Additionally, it is difficult to absorb distance and direction information in unfamiliar environments. Users may make navigation errors or have revisiting behaviors and negative feelings in the exploration of a VE [4,5,6].

Various visual aids that provide spatial knowledge of the environment by graphical means [7] are usable and effective in performing the common VE tasks regarding navigation and object selection/manipulation [8]. Previous studies have indicated that the effectiveness of an overview map is better than wedge, scaled arrows [9], animation guide, and query-based direction information [10] in terms of performing spatial tasks in VEs. With an overview map that show a survey of the environment as a navigation aid, users can sense the spatial layout precisely [11] and do not have to acquire survey knowledge through widest navigation in the VE [12]. Users with an overview map have better sense of direction and security, higher satisfaction, preference, and efficiency than those without an overview map; however, overview maps occupy screen space, add complexity to the system, and require more mental and temporal demand to integrate the distinct views [13,14,15].

In this paper, overview map design supporting navigation in a VE is studied. An experimental study examining the effects of the size and transparency of overview maps on users’ spatial perception and subjective evaluations is conducted. The contribution of our work is composed as follows:

- This is the first work to integrate the visual variables of size and transparency in one study of VE interfaces, making it possible to determine the most appropriate overview map design for user navigation.
- A design principle for better navigation performance: High level of transparency significantly improves efficiency for users with the overview map in the condition of 1/2 screen size. Low level of transparency significantly improves efficiency for users with the overview map in the condition of 1/8 screen size. For the 20% transparent overview map, 1/8 screen size and 1/16 screen size offer significantly higher efficiency than 1/2 screen size.
- The findings from this study suggest that reducing the size of overview maps could effectively improve users’ subjective evaluations of the 3D VE user interfaces.

## 2. Related Work

**Figure 1:**
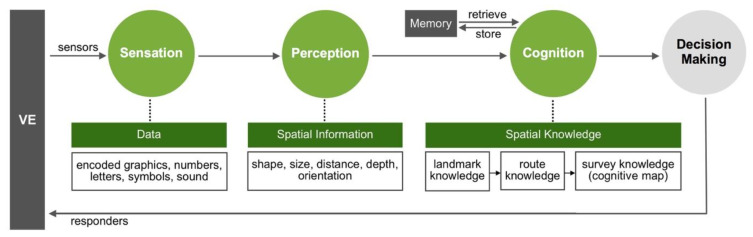
The human information-processing stages regarding the navigation in virtual environments.

Spatial cognition emerges from the interaction between an organism and environmental characteristics [16]. Figure 1 shows human information-processing stages regarding navigation in VEs. Firstly, users pay attention to virtual stimulus such as graphics and text on navigation aids and collected encoded sensory data in several sensory organs. Then spatial information from the various senses and from memory are manipulated or reorganized by cognitive systems [17]. Humans understand the meanings of pivotal landmarks and acquire route knowledge which is integrated by survey knowledge [18,19]. Thus, visual information significantly contributes to forming a cognitive map [20], which represents the spatial environment in the brain [21]. Prior experimental studies have demonstrated significant influence of different visual designs of a navigation aid on user performance and subjective evaluations in VEs. For example, positive landmarks in a VE are more accurate in locating the landmarks and drawing the route as compared to negative and neutral landmarks [16]. The 3D arrows outperform the two-dimensional (2D) arrows in terms of navigation performance and user preference in an abstract desktop VE that did not resemble any unfamiliar environment [12]. The 2D wedge outperform the 3D wedge with respect to navigation performance and interface usability in a VE [22]. Nevertheless, there are currently no specific design guidelines pertinent to overview maps for VE interfaces.

Healey [23] suggested that important information should be displayed with salient visual features, including size, density, hue, luminance, 3D depth and so on, to help draw viewers’ attention. Size as a visual variable has been shown to be more efficient and effective to guide attention than color value, color hue, and orientation in the animated flicker 2D maps [24]. The proverbial limited screen space of an onscreen VE restricts the size of a navigation aid. A large navigation aid occludes many virtual objects, while reducing its size sacrifices the legibility of contents [8]. The size of an overview map is generally smaller than the detail view, and it is rarely the same or larger than the detail view [25]. An early study reviewed 13 studies on the overview and found that the median size of the overview region was 1/10 of the screen space [26]. Users perform worse for higher ratios of overview map size and screen size in navigation and object selection tasks, especially for small screens [26]. Hornbæk et al. [14] suggested that an overview map should at least be 1/16 of the size of a detail view.

Transparency means the variations of color value and saturation [27], and it is frequently expressed as a percentage [28]. Several studies indicated that transparency can solve occlusion issue [8,13,27], draw viewers’ attention [29,30], and help users perform spatial perception tasks in a 3D VE [31,32]. On the contrary, transparency may induce a superposition of different hues or patterns [27] and add a large amount of potentially disturbing complexity, which may compromise the legibility of content [33]. Besides, users using a semi-transparent overview map performed significantly better than those using an opaque overview map regarding a difficult navigation task in VEs [34]. Harrison et al. [35] suggested that the 50% transparent tool palette overlaid on different background content was significantly better than 90% transparent in task performance and legibility.

The above-mentioned studies have shown that the visual variables of size and transparency may play crucial roles in dealing with visibility issue, which is the most prevalent perception issue that can limit users’ experience of a VE interface [8]. The studies on the size or transparency of overview maps in VEs are summarized in Table 1. Altering the size or transparency of an overview map changes the perceptual cues in users’ visual fields. However, studies pertaining to detailed design guidelines on the overview maps in VEs are still insufficient. Thus, the effects of the size and transparency of overview maps on users’ spatial perception and their subjective evaluations of VE interfaces are expected to be assessed in this study.

Users’ spatial perception can be evaluated by performing tasks based on the perceived spatial information about visible objects, which is particularly appropriate for interactive VEs [3]. In the present study, navigation performance with overview maps in a VE is detected using task completion time and error count as primary performance criteria. Additionally, users’ spatial perception and subjective evaluations are closely related [36,37,38]. Some immediate outcomes, including efficiency, subjective satisfaction, subjective preference, and mental workload, can be explicitly measured to subjectively evaluate overview map interfaces [11]. The system usability scale (SUS) [39] has been widely used to evaluate usability, which refers to the effectiveness, efficiency, safety, and enjoyment in interactions with VE interfaces [40,41,42]. Thus, the degree of subjective satisfaction, subjective preference, and system usability was regarded as the constructs of users’ subjective evaluations of the VE interfaces, which were assessed by post-experimental questionnaires in this study.

## 3. Materials and Methods

### 3.1. Experiment Design and Hypotheses

This study employed a between-subjects design involving two variables (the transparency of overview maps and the size of overview maps). Two discrete transparent conditions were tested, i.e., 20% transparent (mostly opaque) and 80% transparent (mostly transparent). The VE was experienced through a screen. The levels of the size of overview maps were 1/2 screen, 1/8 screen, and 1/16 screen. The dependent variables were task completion time and error count, evaluations of satisfaction, preference, and system usability.

Four hypotheses guided the design of the experiment:

- H1: The transparency of overview maps can make significant differences in users’ spatial perception and their subjective evaluations of the 3D VE user interfaces.
- H2: The size of overview maps can make significant differences in users’ spatial perception.
- H3: Users’ subjective evaluations of the 3D VE user interfaces can be improved by reducing the size of overview maps.
- H4: A significant interaction effect exists between the size and transparency of overview maps.

### 3.2. Participants

One hundred and eight university students were recruited as volunteers to participate in the experiment via convenience sampling method, comprising 83 females and 25 males, ranging in age from 17 to 30 (M = 21.44, Sd = 1.35). Each group contained 18 participants, and each participant tested only one kind of overview map:

- Group with the 1/2 screen-size 80% transparent overview map: 13 females and 5 males;
- Group with the 1/8 screen-size 80% transparent overview map: 13 females and 5 males;
- Group with the 1/16 screen-size 80% transparent overview map: 12 females and 6 males;
- Group with the 1/2 screen-size 20% transparent overview map: 17 females and 1 male;
- Group with the 1/8 screen-size 20% transparent overview map: 15 females and 3 males;
- Group with the 1/16 screen-size 20% transparent overview map: 13 females and 5 males.

Twenty-six people had never used overview map interfaces in VEs prior to participating in the experiment. Fifty-seven people had rarely used overview map interfaces in VEs. Twenty-five people had used the interfaces weekly. Ninety-nine percent of participants used smartphones or computers more than two hours per day (M = 6.53, Sd = 3.18, range 0–18 h). All participants used the experimental platform without any problem in basic operation. The study was designed in accordance with the latest version of the Declaration of Helsinki and was approved by the local ethical committee. All participants gave their informed consent for inclusion before they participated in the study.

### 3.3. Prototype and Apparatus

**Figure 2:**
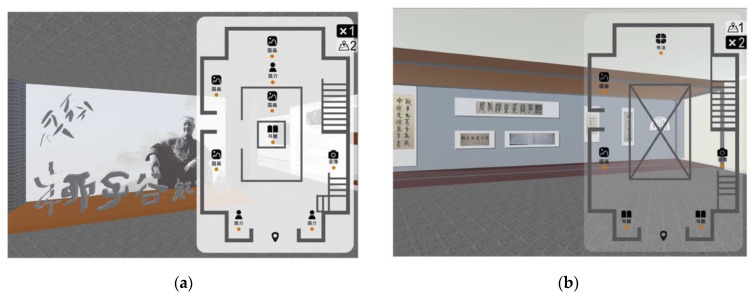
Examples of the virtual museum interfaces: (**a**) the 1/2 screen-size 20% transparent overview map of the ground floor; (**b**) the 1/2 screen-size 80% transparent overview map of the second floor. 简介: Introduction; 国画: Traditional Chinese Painting; 书籍: Books; 影像: Image; 书法: Chinese Calligraphy.

**Figure 3:**
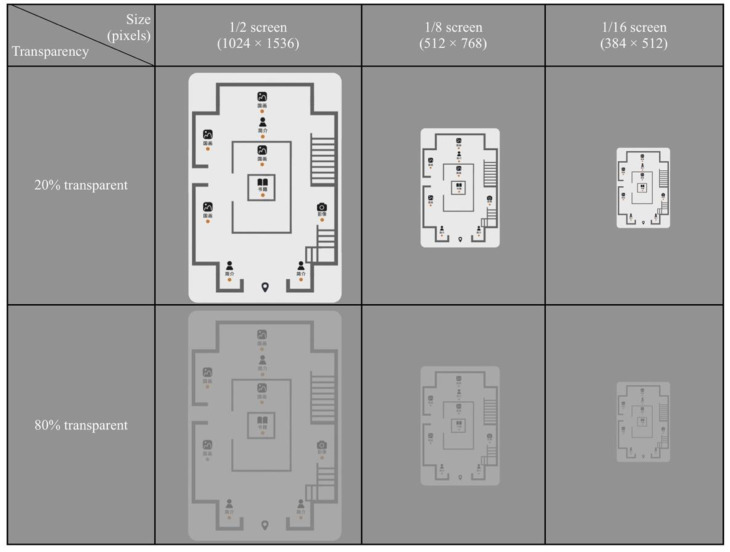
Six types of overview map designs adopted in this study. 简介: Introduction; 国画: Traditional Chinese Painting; 书籍: Books; 影像: Image.

The prototype of VE is created with the 3DS Max software and the Unity 3D game engine. A two-story museum unfamiliar to the participants has been simulated. It consists of nine exhibition areas placed on the ground floor, including four Chinese painting exhibition areas, three introduction exhibition areas, a book exhibition area and a photographic exhibition area. Six exhibition areas are placed around the second floor, which is smaller than the ground floor. There are two Chinese painting exhibition areas, two book exhibition areas, a photographic exhibition area and a Chinese calligraphy exhibition area on the second floor. The overview map interfaces are created with the Photoshop software (see Figure 2). Landmarks with images and text are used to distinguish different exhibition areas. For the experiment, six different overview maps are constructed (see Figure 3). 

The experiment was conducted on an iPad Air tablet computer using the iOS 9.3 operating system in an empty room. The VE interfaces were presented on the 9.7-inch screen with resolution of 2048 × 1536. Our study adopted the first-person perspective which is often used in virtual museums. Visual angles were changed by moving fingers across the screen. To reduce the number of variables affecting user performance, participants were allowed to move in the VE only by clicking the landmark icons on overview maps. Furthermore, there was no other navigation aid in the VE.

### 3.4. Experimental Procedure 

Participants were required to fill out a survey questionnaire about gender, occupation, age, VE navigation experience, and familiarity with computers. Before starting the formal tasks, participants were introduced to the VE user interfaces and allowed to spend as much time as they needed until they felt familiar with the VE. The total duration of this part ranged between 1 and 5 min.

**Figure 4:**
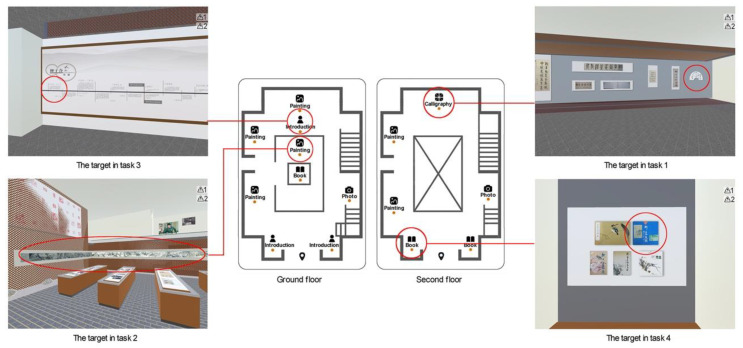
Four spatial perception tasks in this study.

By the method of randomized block design, the participants were randomly assigned to six groups to test one of the six VE user interfaces. The participants were informed that they should perform four spatial perception tasks in sequence as quickly and accurately as possible (see Figure 4). The first task related to shape perception was to find a fan-shaped work in the Chinese calligraphy exhibition area. To complete the task, participants needed to click the floor icons to find the specific exhibition area on the second floor and then change visual angles to perceive the shapes of the virtual objects in the area. The second task concerning size perception was to search for the longest Chinese painting. Participants not only needed to get to six Chinese painting exhibition areas on two floors by clicking the landmark icons, but they also needed to perceive, memorize and compare the sizes of the virtual objects in the areas. The third task related to distance perception was to get the description of artist’s birthday in the farthest introduction exhibition area. Participants needed to compare the distances of three introduction exhibition areas on the ground floor using the overview map and then change positions to look over the details in the specific place. The fourth task about orientation perception was to find out a blue book in the book exhibition area on the west side of the entrance. Participants needed to click the floor icons to find the book exhibition area that met the requirement, and then they found the specific object in it. The participants were asked to explore the environment until they accurately found specific objects in each task. Performance with each overview map was measured in terms of the total time to accomplish four tasks and the total number of errors occurred during this process. An error was scored when the participant found the wrong object. Each error added one point to the participant’s score; otherwise the error count was zero.

After all the tasks had been completed, participants were asked to complete a seven-point Likert scale to rate the overview maps in terms of subjective satisfaction (from 1 “less satisfied” to 7 “very satisfied”) and preference (from 1 “most disliked” to 7 “most liked”). Participants were also required to fill out a SUS questionnaire (see Table 2). Each statement in the ten-question SUS questionnaire was scored using a five-point Likert scale (from 1 “strongly disagree” to 5 “strongly agree”). After the experiment, the participants were asked to do a subjective interview. The interview mainly focuses on overall feelings about the system, causes of errors, and design suggestions. The entire experiment took approximately 15 min. 

## 4. Results

### 4.1. Analyses of Total Task Completion Time and Errors

A 2 x 3 analysis of variance (ANOVA) was performed to analyze the collected data. The results generated from the descriptive statistics and two-way ANOVA of total task completion time are shown in Table 3. It revealed no significant main effect of size in terms of total task completion time (F(2,102) = 2.908, *p* = 0.059 > 0.05). No significant main effect of transparency (F(1,102) = 0.966, *p* = 0.328 > 0.05) was found. Significant interaction existed between the variables of size and transparency (F(2,102) = 6.556, *p* = 0.002 < 0.01).

In order to find out which factor is differentially effective at each level of a second factor, we selected the simple effects test [43]. Table 4 indicates that both overview maps in the condition of 1/2 screen size and 1/8 screen size were significantly different with a transparency factor in total task completion time. It could also be seen that 20% transparent overview maps were significant with a size factor in total task completion time.

**Figure 5:**
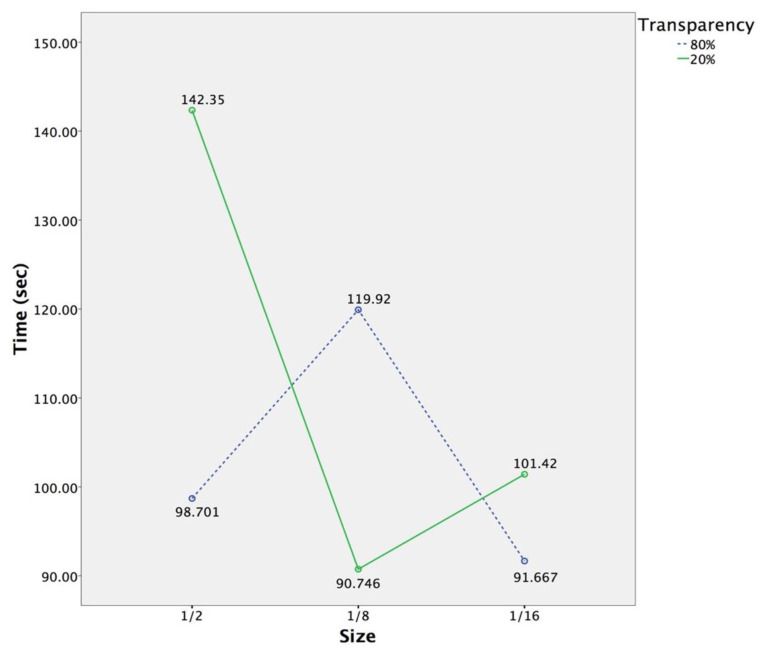
The interaction diagram of the size and transparency of overview maps in terms of total task completion time.

According to Figure 5, at 1/2 screen size, the total task completion time for the 20% transparent overview map (M = 142.354, Sd = 48.451) was significantly longer than that for the 80% transparent overview map (M = 98.701, Sd = 38.449). In contrast, at 1/8 screen size, the total task completion time for the 80% transparent overview map (M = 119.918, Sd = 55.320) was significantly longer than that for the 20% transparent overview map (M = 90.746, Sd = 35.156). For the 20% transparent overview map, participants using overview maps in the condition of 1/16 screen size (M = 101.418, Sd = 43.327) and 1/8 screen size performed significantly better than those using the overview map in the condition of 1/2 screen size. Overall, the total task completion time for the 1/2 screen-size 20% transparent overview map was longer than the others. In addition, participants completed all the tasks with the 1/8 screen-size 20% transparent overview map and the 1/16 screen-size 80% transparent overview map (M = 91.667, Sd = 30.637) similarly more quickly than the others.

A 2 x 3 ANOVA was performed. The results generated from the descriptive statistics and two-way ANOVA of total errors occurred in all the tasks is shown in Table 5. No significant main effect of size (F(2,102) = 0.757, *p* = 0.471 > 0.05) or transparency (F(1,102) = 0.000, *p* = 1.000 > 0.05) was found. There existed no significant interaction effect between the variables of size and transparency (F(2,102) = 0.757, *p* = 0.471 > 0.05). Participants in each group made few mistakes.

### 4.2. Analysis of Subjective Satisfaction

Satisfaction refers to users’ experience of having positive feelings, such as confidence and control, in their performance of tasks. After completing the tasks, comparisons and analysis of subjective satisfaction were made using a 2 x 3 ANOVA. The descriptive statistics and two-way ANOVA of satisfaction are shown in Table 6. In this experiment, the mean value of satisfaction in each group was over 5, and the mean value of overall satisfaction (M = 5.81, Sd = 0.877) indicates that the overview maps were inclined to be satisfactory.

The results regarding subjective satisfaction reveals a significant main effect of size (F(2,102) = 4.656, *p* = 0.012 < 0.05). The results of post hoc comparison using the least significant difference (LSD) show a significant difference between the condition of 1/8 screen size and 1/2 screen size (*p* = 0.003 < 0.01). The satisfaction score of the overview map in the condition of 1/8 screen size (M = 6.14, Sd = 0.683) was significantly higher than that of the overview map in the condition of 1/2 screen size (M = 5.53, Sd = 0.878). No significant difference was found in the main effect of transparency (F(1,102) = 0.203, *p* = 0.653 > 0.05). There was no significant interaction effect between the variables of size and transparency (F(2,102) = 0.622, *p* = 0.539 > 0.05).

### 4.3. Analysis of Subjective Preference

After the tasks were completed, participants were asked to rate how much they like the interface to recognize the most popular overview map. A 2 x 3 ANOVA was performed. The results generated from the descriptive statistics and two-way ANOVA of preference are shown in Table 7. The mean value of preference in each group was more than 4.8, and the mean value of the overall preference (M = 5.62, Sd = 1.039) shows that participants like to use the VE user interface.

The results regarding subjective preference reveal that there was a significant main effect of size (F(2,102) = 5.044, *p* = 0.008 < 0.01). The results of post hoc comparison using the LSD show a significant difference between the condition of 1/8 screen size and 1/2 screen size (*p* = 0.007<0.01). The condition of 1/16 screen size and 1/2 screen size also show a significant difference (*p* = 0.007 < 0.01). The preference scores of the overview map in the condition of 1/8 screen size (M=5.83, Sd=0.878) and 1/16 screen size (M = 5.83, Sd = 0.910) were significantly higher than that of the overview map in the condition of 1/2 screen size (M = 5.19, Sd = 1.191). No significant main effect of transparency (F(1,102) = 0.467, *p* = 0.496 > 0.05) was found. The interaction effect between the variables of size and transparency was significant (F(2,102) = 3.156, *p* = 0.047 < 0.05).

Based on the simple effect test results (Table 8), the overview maps in the condition of 1/2 screen size were significantly different with a transparency factor in terms of preference. It could also be seen that 20% transparent overview maps were significant with a size factor in terms of preference.

**Figure 6:**
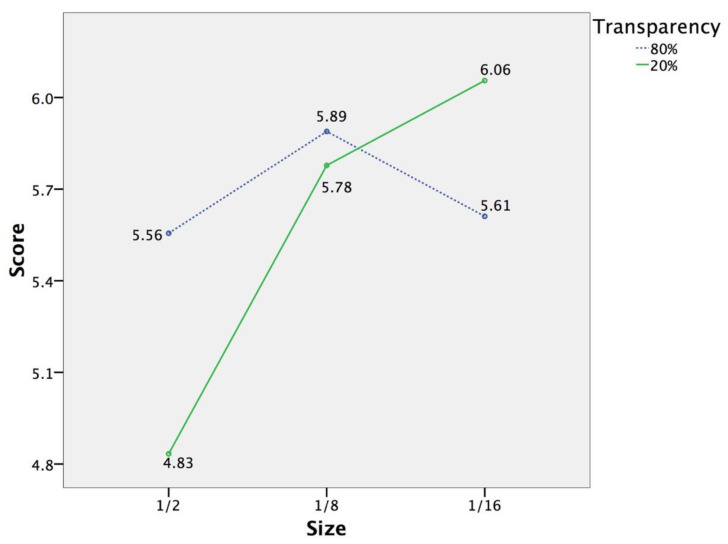
The interaction diagram of the size and transparency of overview maps in terms of preference.

According to the interaction diagram illustrated in Figure 6, at 1/2 screen size, the preference score of the 80% transparent overview map (M = 5.56, Sd = 1.247) was significantly higher than that of the 20% transparent overview map (M = 4.83, Sd = 1.043). For the 20% transparent overview map, participants using overview maps in the condition of 1/16 screen size (M = 6.06, Sd = 0.873) and 1/8 screen size (M = 5.78, Sd = 0.647) gave significantly higher preference scores than those using the overview map in the condition of 1/2 screen size. Overall, the preference score of the 1/2 screen-size 20% transparent overview map was lower than the others. In addition, the preference score of the 1/16 screen-size 20% transparent overview map was higher than the others.

### 4.4. Analysis of SUS

A 2 × 3 ANOVA was performed. The generated results of the SUS questionnaire are shown in Table 9. The mean value of SUS (M = 74.907, SD = 11.661) shows that the usability of VE user interfaces was good and the overview maps were easy to use for participants. The ANOVA results point out that there was a significant main effect of size (F(2,102) = 3.673, *p* = 0.029 < 0.05). The results of post hoc comparison using the LSD show a significant difference between the condition of 1/2 screen size and 1/8 screen size (*p* = 0.008 < 0.01). Participants using the overview map in the condition of 1/8 screen size (M = 78.194, Sd = 11.062) gave significantly higher SUS scores than those using the overview map in the condition of 1/2 screen size (M = 71.042, Sd = 11.625). It revealed no significant main effect of transparency (F(1,102) = 1.224, *p* = 0.271 > 0.05). No significant interaction effect was found between the variables of size and transparency (F(2,102) = 1.625, *p* = 0.202 > 0.05).

### 4.5. Qualitative Results

Besides the quantitative results, we also observed the process in which the participants interacted with the VE user interfaces, and we collected qualitative data from post interviews. The results are clearly positive: none of the participants had difficulties in using the experimental apparatus. All participants were satisfied with the simple operation of the system. Three participants who tried to zoom in on the overview map in the condition of 1/16 screen size commented that the contents of the overview map are a little hard to be recognized. None of the participants with overview maps in the condition of 1/8 screen size or 1/16 screen size tried to hide the overview map. Two participants reported that one of the difficulties in performing the tasks was the rapid change in visual angle.

## 5. Discussion

The analysis of total task completion time confirmed the fourth hypothesis that significant interaction effect exists between the size and transparency of overview maps. At 1/2 screen size, participants using the 80% transparent overview map performed significantly better than those using the 20% transparent overview map. At 1/8 screen size, participants using the 20% transparent overview map performed significantly better than those using the 80% transparent overview map. For the 20% transparent overview map, participants using overview maps in the condition of 1/16 screen size and 1/8 screen size performed significantly better than those using the overview map in the condition of 1/2 screen size. This might be because the 1/2 screen-size overview map and the 20% transparent overview map reduce the visibility and legibility of VE contents. Spatial perception tasks might be difficult when some relevant objects and locations are invisible. Smaller overview maps taking less screen space from the VE interface, and more transparent overview maps, allowing more underlying layer data to pass through them [28], can both display more spatial information about virtual objects. For the smaller overview map in the condition of 1/8 screen size, one possible explanation is that distracting visual cues from underlying opaque layer interact with the overview map, increasing the similarity between objects belong to different layers [28] and decreasing the efficiency of visual search [23]. In line with earlier studies [33], a high level of transparency compromises the legibility of map contents and eliminate users’ cognitive load of processing spatial relationships. The results from total task completion time are in accordance with previous studies suggesting that the size and transparency of overview maps have impacts on displayed information contents, users’ cognitive mapping and navigation performance [14,25,34]. The results indicate that the effects of the transparency of overview maps on users’ spatial perception in 3D VEs vary with the size of overview maps. Contrary to the first and second hypotheses, the size and transparency of overview maps cannot make significant differences in users’ spatial perception.

Concerning subjective satisfaction, the results confirmed the third hypothesis that users’ subjective evaluations of the 3D VE user interfaces can be improved by reducing the size of overview maps. The overview map in the condition of 1/8 screen size led to significantly higher user satisfaction compared to the condition of 1/2 screen size. This might be because the overview map in the condition of 1/8 screen size provides more details of the VE to make users feel easier to know the environment well. Another aspect that might explain the difference is that the overview map in the condition of 1/2 screen size, which is often deliberately hidden to avoid occlusion in the VE, makes the problem of focus switching worse [8]. 

The results regarding subjective preference confirmed the third hypotheses as well. Users’ subjective preference could be improved by reducing the size of overview maps. Users liked overview maps in the condition of 1/8 screen size and 1/16 screen size significantly better than those in the condition of 1/2 screen size, especially for the 20% transparent overview map. It might be that the overview map in the condition of 1/2 screen size masks more detailed spatial information that users may be interested in. Moreover, it is easier to switch attention between the layers of the VE and a small overview map. Similar to the results from total task completion time, the results regarding subjective preference also confirmed the fourth hypothesis that significant interaction effect exists between the size and transparency of overview maps. At 1/2 screen size, users liked the 80% transparent overview map significantly better than the 20% transparent overview map. Perhaps at 1/2 screen size, a high level of transparency effectively relieves the occlusion. This is consistent with previous studies that propose that making use of transparency influences user preference in navigation tasks [44], and a high level of transparency improves subjective evaluations for users with the larger interface in close proximity [45].

The results from the SUS questionnaire are consistent with those from users’ subjective evaluations of satisfaction and preference, and also confirmed the third hypothesis that users’ subjective evaluations of the 3D VE user interfaces can be improved by reducing the size of overview maps. The usability of the overview map in the condition of 1/8 screen size is considered significantly better than that of the overview map in the condition of 1/2 screen size. Perhaps this is due to the additional steps needed to display or hide the overview map in the condition of 1/2 screen size. It is difficult to switch attention between the layers of the detail view and a large overview map. The results are in accordance with previous studies suggesting that more mental and temporal demand is required to integrate the distinct views for understanding spatial relationships [8,14].

Maps, a predominant form of navigation aid [17], need to be investigated in depth and in detail for VE usage. To advance users’ spatial awareness, overview maps have to deal with occlusion and legibility issues, while conveying correct spatial relations in various conditions of spatial manipulation. A variety of factors may influence users’ action tendencies and feelings during the interaction with the user interfaces of overview maps. In this study, we focus on the design of overview maps on 3D VE user interfaces to provide design suggestions for the development of VE applications. Besides the primary independent variables, other potential factors, such as characteristics of the environment, user, task, and VE design, could be included. The findings are possibly limited by the simple VE tasks. Moreover, the experiment was conducted on a tablet, thus the limitations of screen size and input style should be considered. Participants’ comments, suggesting that the availability of zooming on overview maps could be helpful for navigation in VEs, can be an interesting research issue for future studies. This study is limited in scope, which suggests that further research may include screen size, interacting style, the number of virtual objects, users’ age, and spatial ability.

## 6. Conclusions

This study mainly explored how the size and transparency of overview maps affect navigation in a 3D VE. One hundred and eight participants spent an average of 15 min solving spatial perception tasks using an overview map. Our results suggest that significant interaction effect exists between the size and transparency of overview maps in users’ spatial perception and subjective evaluations of the 3D VE user interfaces. At 1/2 screen size, users with the 80% transparent overview map have significantly higher efficiency and preference than those with the 20% transparent overview map. At 1/8 screen size, the 20% transparent overview map is significantly more efficient than the 80% transparent overview map. For the 20% transparent overview map, users with the overview maps in the condition of 1/16 screen size and 1/8 screen size have significantly higher efficiency and preference than those with the overview map in the condition of 1/2 screen size. Based on our work, we recommend that designers reduce the size of overview maps in navigating onscreen 3D VEs. The overview map in the condition of 1/8 screen size leads to significantly higher subjective evaluations of satisfaction, preference, and system usability than that in the condition of 1/2 screen size. Whether or not the results are consistent using immersive virtual devices and more natural navigation metaphors were not confirmed in this study. As a future work, we will investigate and extend our research to more complex tasks and more immersive VEs.

## Figures and Tables

**Table 1:** Overview map studies on size or transparency.

Paper	Conditions	Tasks	Results
Hornbæk et al. [14]	Interfaces with and without an overview on desktop.	Search for specific objects. Spatial recall of map objects.	• Overview maps should at least be 1/16 of the size of a detail view and should be larger to support navigation for small screens. • Faster navigation and better spatial recall of map objects for interfaces without an overview when the detail interface provides richer cues. • Higher preference for interfaces with an overview. • Users take more time to switch between overview and detail views.
Burigat and Chittaro [25]	Overview maps with and without highlighted objects of interest on mobile devices.	Search for specific objects. Spatial recall of map objects.	• The size of an overview map is generally smaller than the detail view, and it is rarely the same or larger than the detail view. • Both direct manipulation of the overview and highlighting objects of interest in the overview improve navigation performance, but do not help spatial recall of map objects.
Jakobsen and Hornbæk [26]	Focus + context, overview + detail, and zooming interfaces on different displays:0.17, 1.5, and 13.8 megapixels.	Select and compare specific objects. Trace a route.	• The median size of the overview region was 1/10 of the screen space. • Users perform worse for higher ratios of overview map size and screen size in navigation and object selection tasks, especially for small screens.
Chen, M.X. and Chen, C.H. [34]	Opaque and semi-transparent overview maps on tablet devices.	Search for and compare specific objects.	• User performance with a semi-transparent overview map is significantly better than an opaque overview map regarding a difficult navigation task in VEs.

**Table 2:** Likert Scale items of system usability scale (SUS) questionnaire.

No.	Question
1	I think that I would like to use this interface frequently.
2	I found the interface unnecessarily complex.
3	I thought the interface was easy to use.
4	I think that I would need the support of a technical person to be able to use this interface.
5	I found the various functions in the interface were well integrated.
6	I thought there was too much inconsistency in this interface.
7	I imagine that most people would learn to use this interface very quickly.
8	I found the interface very awkward to use.
9	I felt very confident using the interface.
10	I needed to learn a lot of things before I could get going with this interface.

**Table 3:** Descriptive statistics and two-way ANOVA of total task completion time.

Total Task Completion Time	80% Transparent		20% Transparent		Mean (Size)		Size	Transparency	Size × Transparency
	Mean	(SD)	Mean	(SD)	Mean	(SD)	*p*	*p*	*p*
1/2 screen	98.701	38.449	142.354	48.451	120.528	48.459	*p* > 0.05	*p* > 0.05	*p* < 0.01
1/8 screen	119.918	55.320	90.746	35.156	105.332	48.017			
1/16 screen	91.667	30.637	101.418	43.327	96.543	37.312			
Mean (transparency)	103.429	43.632	111.506	47.494					

**Table 4:** Simple effect of total task completion time.

**Size**	**Transparency(I)**	**Transparency(J)**	**MD(I-J)**	**SE**	***P***
1/2 screen	20% transparent	80% transparent	43.653	14.232	*p* < 0.01
1/8 screen	20% transparent	80% transparent	29.172	14.232	*p* < 0.05
1/16 screen	20% transparent	80% transparent	9.752	14.232	*p* > 0.05
**Transparency**	**Size(I)**	**Size (J)**			
80% transparent	1/2 screen	1/16 screen	7.034	14.232	*p* > 0.05
	1/8 screen	1/2 screen	21.217	14.232	*p* > 0.05
	1/8 screen	1/16 screen	28.251	14.232	*p* > 0.05
20% transparent	1/2 screen	1/8 screen	51.608	14.232	*p* < 0.01
	1/2 screen	1/16 screen	40.936	14.232	*p* < 0.05
	1/16 screen	1/8 screen	10.672	14.232	*p* > 0.05

MD = mean difference, SE = standard error.

**Table 5:** Descriptive statistics and two-way ANOVA of total errors.

Total Errors	80% Transparent		20% Transparent		Mean (Size)		Size	Transparency	Size × Transparency
	Mean	(SD)	Mean	(SD)	Mean	(SD)	*p*	*p*	*p*
1/2 screen	0.44	0.705	0.28	0.461	0.36	0.593	*p* > 0.05	*p* > 0.05	*p* > 0.05
1/8 screen	0.44	0.616	0.61	0.502	0.53	0.560			
1/16 screen	0.44	0.616	0.44	0.511	0.44	0.558			
Mean (transparency)	0.44	0.634	0.44	0.502					

**Table 6:** Descriptive statistics and two-way ANOVA of satisfaction.

Satisfaction	80% Transparent		20% Transparent		Mean (Size)		Size		Transparency	Size × Transparency
	Mean	(SD)	Mean	(SD)	Mean	(SD)	*p*	Post Hoc (LSD)	*p*	*p*
1/2 screen	5.67	1.029	5.39	0.698	5.53	0.878	*p* < 0.05	1/8 > 1/2	*p* > 0.05	*p* > 0.05
1/8 screen	6.06	0.802	6.22	0.548	6.14	0.683				
1/16 screen	5.83	0.985	5.72	0.958	5.78	0.959				
Mean (transparency)	5.85	0.940	5.78	0.816						

**Table 7:** Descriptive statistics and two-way ANOVA of preference.

Preference	80% Transparent		20% Transparent		Mean (Size)		Size		Transparency	Size × Transparency
	Mean	(SD)	Mean	(SD)	Mean	(SD)	*p*	Post Hoc (LSD)	*p*	*p*
1/2 screen	5.56	1.247	4.83	1.043	5.19	1.191	*p* < 0.01	1/8 > 1/2, 1/16 > 1/2	*p* > 0.05	*p* < 0.05
1/8 screen	5.89	1.079	5.78	0.647	5.83	0.878				
1/16 screen	5.61	0.916	6.06	0.873	5.83	0.910				
Mean (transparency)	5.69	1.079	5.56	1.003						

**Table 8:** Simple effects test for preference.

**Size**	**Transparency(I)**	**Transparency(J)**	**MD(I-J)**	**SE**	***P***
1/2 screen	80% transparent	20% transparent	0.722	0.328	*p* < 0.05
1/8 screen	80% transparent	20% transparent	0.111	0.328	*p* > 0.05
1/16 screen	20% transparent	80% transparent	0.444	0.328	*p* > 0.05
**Transparency**	**Size(I)**	**Size (J)**			
80% transparent	1/8 screen	1/2 screen	0.333	0.328	*p* > 0.05
	1/8 screen	1/16 screen	0.278	0.328	*p* > 0.05
	1/16 screen	1/2 screen	0.056	0.328	*p* > 0.05
20% transparent	1/8 screen	1/2 screen	0.944	0.328	*p* < 0.05
	1/16 screen	1/2 screen	1.222	0.328	*p* < 0.01
	1/16 screen	1/8 screen	0.278	0.328	*p* > 0.05

MD = mean difference, SE = standard error.

**Table 9:** Descriptive statistics and two-way ANOVA of SUS

SUS	80% Transparent		20% Transparent		Mean (Size)		Size		Transparency	Size × Transparency
	Mean	(SD)	Mean	(SD)	Mean	(SD)	*p*	Post Hoc (LSD)	*p*	*p*
1/2 screen	74.03	12.61	68.06	10.02	71.04	11.62	*p* < 0.05	1/8 > 1/2	*p* > 0.05	*p* > 0.05
1/8 screen	76.67	12.37	79.72	9.70	78.19	11.06				
1/16 screen	77.64	13.13	73.33	9.39	75.49	11.46				
Mean (transparency)	76.11	12.56	73.70	10.67						
